# Revisiting *Membranes*—An Open Access Membrane Science and Technology Journal

**DOI:** 10.3390/membranes14040093

**Published:** 2024-04-19

**Authors:** Spas D. Kolev

**Affiliations:** School of Chemistry, The University of Melbourne, Melbourne, VIC 3010, Australia; s.kolev@unimelb.edu.au

*Membranes* is celebrating its 13th anniversary this year. Since its inaugural issue that was released in 2011 [1], it has established itself as a reputable platform where original membrane research can be presented and discussed with minimal delay.

In 2019, *Membranes* was indexed in Science Citation Index Expanded (SCIE)—Web of Science (Clarivate Analytics)—and shortly after received its first CiteScore of 2.69 for the previous year, which has increased since then to 4.4 (for 2023). In 2020, *Membranes* received is first Impact Factor of 3.094 (for 2019), which is now a respectable 4.2 (for 2023). Also, in 2020, *Membranes* published its 1000th paper.

Throughout its existence, *Membranes* has proudly supported the membrane research community with a range of activities involving the development of affiliate relationships with membrane societies (e.g., European Membrane Society, Membrane Society of Australasia, and Polish Membrane Society), providing funding to conferences where membrane research is reported, publishing conference and special issues, and establishing the Best Paper Award, to name a few. Special attention has been paid to the professional development of early career membrane researchers by offering travel support and establishing the Young Investigator Award.

In its early years, *Membranes* focused mainly on research involving non-biological membranes with an emphasis on porous membranes because of their widespread use in important commercial applications, such as desalination of seawater and brackish water and the clean-up of industrial effluents. However, research on non-porous membranes, such as liquid membranes (e.g., supported liquid membranes, emulsion liquid membranes, bulk liquid membranes, and polymer inclusion membranes), which have bridged the gap between membrane separation and solvent extraction, has also been prominently featured.

Research on biological membranes, which are critical to all forms of life, has exploded in recent years. Moreover, applications of such membranes have also become of increasing interest to industry and have been extensively covered in recent years by *Membranes*.

*Membranes* now covers research in the broad areas of (1) biological membranes, (2) membrane fabrication and characterization, (3) membrane applications (water treatment), (4) membrane applications (energy), (5) membrane applications (gas separation), and (6) membrane applications (other areas).

The section “Biological Membranes” covers all aspects of biological membrane research, including both fundamental life science research and the industrial applications of biological membranes. The “Membrane Fabrication and Characterization” section focuses on important features and on the in-depth understanding of membrane fabrication methods and characterization techniques. The four sections on membrane applications publish articles reporting on cutting-edge membrane research in established and emerging industries where membrane processes play a critical role.

More details about these sections and the specific topics they cover can be found under the “Aims and Scope” section.

Authors are invited to submit manuscripts that contribute to the advancement of the various aspects of membrane science covered in the abovementioned sections.
